# SG-APSIC1050: Personal care formulations prove effective against evolving variants of SARS-CoV-2: Implications for public health and hygiene

**DOI:** 10.1017/ash.2023.46

**Published:** 2023-03-16

**Authors:** Sayandip Mukherjee, Carol K Vincent, Harshinie W Jayasekera, Ashish S Yekhe

**Affiliations:** Unilever Industries Private Limited, Mumbai, India; Unilever Research & Development, Trumbull, Connecticut, United States; Unilever Sri Lanka, Colombo, Sri Lanka; Hindustan Unilever Limited, Mumbai, India

## Abstract

**Objectives:** Early in the COVID-19 pandemic, global health authorities identified and emphasized the importance of practicing proper hand hygiene to reduce the transmission of SARS-CoV-2 and to diminish the chances of becoming infected. It is well established in the scientific literature that surfactants and alcohols are capable of inactivating enveloped viruses such as SARS-CoV-2. However, given the novel nature of the virus, Unilever adopted an evidence-based approach to demonstrate virucidal efficacy of marketed bar soaps, liquid handwashes, and alcohol-based hand sanitizers against the original and selected variants of SARS-CoV-2. **Methods:** High titers of clinically isolated and laboratory-propagated SARS-CoV-2 strains were subjected to a range of selected proprietary formulations from Unilever at end-user–relevant dilutions, temperature, and contact duration, and were tested according to the internationally recognized ASTM E-1052 test protocol. **Results:** All tested personal-care formulations were effective against the parental SARS-CoV-2 strain as well as the β (beta) and δ (delta) variants of concern. More specifically, bar soaps with a varying concentration of total fatty matter content and liquid handwashes with varying levels of total surfactants reduced the viral titer by >99.9% within 20 seconds. Alcohol-based hand sanitizers demonstrated >99.99% reduction of input viral load within 15 seconds of contact with the viral inoculum. **Conclusions:** In conclusion, we have provided empirical proof that well-designed personal-care formulations that act through generic physicochemical mechanism against the basic structure of the virus particle have high virucidal efficacy against the original and evolved SARS-CoV-2 variants. Furthermore, we argue that due to the broad-spectrum mode of action of these tested formulations, the continued practice of good hand hygiene practices with everyday products holds significant promise as an easily accessible, economic, and effective nontherapeutic public health intervention toward reducing the transmission of present and future variants of SARS-CoV-2 across communities and populations.

